# Supplementary material for: Elevated carbon monoxide observations over the North Pacific upper troposphere (July 2012–February 2013)
Source: PLoS One. 2025 May 21;20(5):e0323460. doi: 10.1371/journal.pone.0323460 (PMC12094778; doi:10.1371/journal.pone.0323460)
Supplement: Attachment — Submitted filename: Response_to_Reviewers.pdf [file pone.0323460.s001.pdf]

Dear Editor Dr. Markus Metsälä,

Thank you for providing the opportunity to revise our manuscript (PONE-D-24-32569) entitled "In-Situ Observation and Attribution of Elevated Carbon Monoxide Over the North Pacific Upper Troposphere from Regular In-Service Commercial Passenger Airplanes During July 2012 - February 2013." We appreciate the constructive feedback provided by the reviewers and yourself, and we have made comprehensive revisions to address the comments raised. Below, we provide a detailed, point-by-point response to each comment.

The funders had no role in study design, data collection and analysis, decision to publish, or preparation of the manuscript.

Reviewer #1 Comments:

General Comments:

- **Comment:** The manuscript is well done and provides high-quality analysis. However, the title seems too long.
    - **Response:** Thank you for the positive feedback. We have shortened the title to: "Elevated Carbon Monoxide Observations Over the North Pacific Upper Troposphere (July 2012 - February 2013)."
- **Comment:** The manuscript could be written more concisely, and some figures could be combined or removed.
    - **Response:** We have revised the manuscript to streamline the text. Figures 4 shows a typical flight and the collection of data used in this work. We have revised Figure 5 and Figure 7 to increase clarity of the figure.

Specific Comments:

- **Comment (L204):** Remove the "?", also in other places throughout the text (e.g., L367).
    - **Response:** We have removed the "?" and conducted a thorough check to ensure such typographical issues are corrected throughout the manuscript.
- **Comment (L237):** This sentence is incomplete.
    - **Response:** We have revised the sentence for clarity and completeness.
- **Comment (L301):** Remove this line.
    - **Response:** The specified line has been removed.

- **Comment (Fig. 4 caption):** Add the date; upper-left panel: add "hour" to the x-axis label; lower-left panel: add the name and unit for the color bar.
    - **Response:** These adjustments have been made in the figure caption and the figure itself.
- **Comment (Fig. 5):** Provide a mean profile in each panel.
    - **Response:** Mean profiles have been added to each panel of Figure 5.
- **Comment (Section 3.2.4, Fig. 6):** Fix references to Fig. 6d and 6e, which were discussed but are missing.
    - **Response:** Figures 6d and 6e have been added, and the caption has been revised to match the panels.
- **Comment (Fig. 7):** Improve the quality of the plots.
    - **Response:** The resolution and clarity of Figure 7 have been improved.
- **Comment (Fig. 8):** Ensure all panels are the same size and indicate the limits for elevated and low CO in the caption.
    - **Response:** Figure 8 has been redrawn with uniform panel sizes, and the caption now includes CO limits. The lowest panel contains 2-digit numbers in the color bar, which slightly reduce the figure size when compared with above two panels which contains 3-digit numbers in the color bar.
- **Comment (Fig. 9 caption):** Correct "month" (previously misspelled as "mont").
    - **Response:** The typo has been corrected.
- **Comment (Fig. 12):** please explain the color bars in the caption.
    - **Response:** We have revised description of the color bars in the caption.
- **Comment (Fig. 14):** caption, should elevated CO cases be in upper panels, against low CO cases in lower panels (not left and right panels written in the caption)? Please clearly indicate and differentiate the two color bars (name and unit). Please also indicate the definitions of high and low CO limits.
    - **Response:** We have revised the description of figure caption, stated the definition of high and low CO limits (as also described in section 2.3.
- **Comment (Section 3.2.4):** Provide additional figures or tables to compare differences in CO for the same season and latitude/longitude regions.
    - **Response:** We have revised Figure 7 so that differences in CO for the same month can be compared for the same season. We have also included statistical box charts in the figure so that the mean, maximum, minimum, 25th, 50th and 75th percentiles of measurements can be compared. We have also included month in each subplot to increase the clarity of the plots for comparisons.
- **Comment (Section 3.2.4):** Fig. 6 contains no Fig. 6e and 6d, which was discussed in the text.

- **Response:** We have revised Figure 6, and included Figure 6e and 6d in the revised Figure 6.

Reviewer #2 Comments:

General Comments:

- **Comment:** The manuscript might be better suited for journals like JGR-Atmospheres or ACP.
  - **Response:** Thank you for the suggestion. While we appreciate the recommendation, we believe PLOS ONE's inclusive scope is appropriate for our multidisciplinary approach.
- **Comment:** The manuscript is lengthy and includes excessive details. Focus on key findings and remove unnecessary content.
  - **Response:** We have significantly revised the figures to increase the clarity of the manuscript.
- **Comment:** Support findings with a trend analysis of NOAA flask measurements of CO.

Response: Thank you for the suggestion. A trend analysis using NOAA flask measurements has been addressed in a separate manuscript (https://egusphere.copernicus.org/preprints/2024/egusphere-2024-2414/). To provide additional context in this manuscript, we have revised Figure 7 to include a statistical comparison of CO measurements from the NASA GTE experiments and IAGOS data. The revised figure presents box charts showing the mean, maximum, minimum, and the 25th, 50th (median), and 75th percentiles of CO concentrations for corresponding seasons (months).

From this comparison:

- **PEM-West A (1991)**: Mean CO concentrations are around 100 ppbv, with occasional elevated CO concentrations exceeding 200 ppbv at altitudes below 4 km.
- **IAGOS Measurements (2012)**: Mean CO concentrations are higher than 100 ppbv, with elevated CO frequently exceeding 200 ppbv throughout the troposphere.

These findings highlight the temporal changes in CO concentrations over two decades and underscore the differences between the historical NASA GTE experiments and more recent IAGOS measurements.

Specific Comments:

- **Comment (Line 48):** Do you really mean 'surface emissions'? Do you mean concentrations?
  - **Response:** We have revised the text to specify "CO concentrations"
- **Comment (Section 2.2 header):** Correct "Te" to "The."
  - **Response:** The typo has been corrected.
- **Comment (Section 3.1.1 and 3.1.2 headers):** Correct "flght" to "flight."
  - **Response:** The typo has been corrected.
- **Comment (Fig. 5):** Label regions (e.g., "Asian Industrial" for Fig. 5d).
  - **Response:** Labels have been added to Figure 5.
- **Comment (Lines 209-217):** Figures 5e and 5f are missing.
  - **Response:** Figures 5e and 5f have been added and referenced appropriately.
- **Comment (Line 237):** Unfinished sentence.
  - **Response:** We have completed the sentence.
- **Comment (Fig. 7):** This figure is a little difficult to read. I would suggest making median profiles (with interquartile range horizontal bars) to compare IAGOS with field campaigns on the same plot. If you want to show that CO has increased, it could be helpful to show probability distributions from each campaign.
  - **Response:** we have revised Figure 7 to include a statistical comparison of CO measurements from the NASA GTE experiments and IAGOS data. The revised figure presents box charts showing the mean, maximum, minimum, and the 25th, 50th (median), and 75th percentiles of CO concentrations for corresponding seasons (months).
- **Comment (Section 3.3):** Are you trying to answer the question of tropospheric distributions alone? Or including the influx of stratospheric air? It might be useful to filter out stratospheric air (say with a filter [O3] / [CO] > 1.25 mol mol−1). Then you could also talk about the amount of stratospheric influence in each region or time period.
  - **Response:** Thank you for your insightful comment. In this study, we focus solely on addressing the distribution of elevated CO in the troposphere and do not include the influence of stratospheric air. While filtering for stratospheric contributions (e.g., using the [O_3]/[CO] > 1.25 mol mol^{-1} criterion) is indeed a valuable approach, such analyses are beyond the

scope of this work. However, the influence of stratospheric air on tropospheric composition, including ozone and CO distributions, has been explored in a separate study:
Wang, K.Y., Kau, W.S. (2015). "Simulation of impact from stratospheric ozone on global tropospheric ozone distribution with a chemistry transport model: A case study during the 1990–1991 period." *Asia-Pacific Journal of Atmospheric Sciences*, 51, 137–155. https://doi.org/10.1007/s13143-015-0064-7.

- We appreciate your suggestion and recognize the importance of stratospheric-tropospheric exchange (STE) in shaping tropospheric composition. Future studies could integrate such filtering techniques to better quantify the extent of stratospheric influence in different regions and time periods.

- **Comment (Lines 313-314):** Are you saying there is also convective activity in September and October? Or is there a different explanation?
  - **Response:** Thank you for this insightful question. Yes, there is convective activity during September and October over East Asia, albeit with different characteristics compared to the peak summer monsoon season. This convective activity can play a significant role in the vertical transport of air pollutants. **Convective Activity in September and October:** Studies highlight that the Asian Summer Monsoon Anticyclone (ASMA) persists into early autumn, extending through September and, to a lesser extent, October. This system is associated with strong convective activity in regions spanning 70°–120°E, including East Asia. Research on the ASMA (e.g., Wright et al., 2011) emphasizes its role in lifting surface emissions, such as carbon monoxide (CO) and other pollutants, from East Asia to higher altitudes during this time. **Seasonal Transition and Post-Monsoon Convection:** During September and October, the monsoon begins to retreat, and post-monsoon convection becomes less widespread but is still active in localized regions, particularly over East Asia. This transitional period is characterized by regional convective events that can continue to transport pollutants vertically. **Comparison with November to February:** Convective activity decreases significantly from November to February due to the onset of the dry season and cooler temperatures. However, during these months, "cold surges" and associated frontal systems over East Asia can enhance vertical mixing and pollutant transport. Studies such as Liu et al. (2003) show that these cold surges can transport East Asian

pollutants equatorward, where they may be further lifted by localized convection in the tropics. **Pollutant Transport by Convection:** During both periods, convection facilitates the vertical transport of surface-emitted pollutants, including CO, to the upper troposphere. For example:

- **September – October:** Convective events associated with the weakening ASMA contribute to pollutant uplift.
- **November – February:** Cold surge-induced frontal activity and tropical convection play a more dominant role in pollutant transport.

In summary, the observed elevated CO concentrations in September and October can be attributed to convective activity associated with the retreating ASMA and localized convection. This contrasts with the colder months (November – February), when cold surges and associated frontal systems drive pollutant transport. These processes highlight the dynamic interplay between seasonal meteorology and pollutant distribution over East Asia.

- **Wright et al. (2011):**

Wright, J. S., Fueglistaler, S., & Wexler, A. S. (2011). Variability of convective activity in the tropical tropopause layer. *Journal of Geophysical Research: Atmospheres*, 116(D12), D12103.
DOI: 10.1029/2011JD016337

- **Liu et al. (2003):**

Liu, J., Mauzerall, D. L., Horowitz, L. W., Fiore, A. M., & Naik, V. (2003). Air pollution control policies in China: Recent developments and future challenges. *Journal of Geophysical Research: Atmospheres*, 108(D20), 8797.
DOI: 10.1029/2002JD003178

- **Characteristics of convection and advection associated with the Asian Summer Monsoon Anticyclone (ASMA):**

This study highlights the persistence of the ASMA into September and its role in lifting pollutants to higher altitudes.
Source: *Climate Dynamics*, DOI: 10.1007/s00382-024-07371-3

- **Transport of Asian surface pollutants to the global stratosphere from the Tibetan Plateau region during the Asian summer**

monsoon:
Focuses on the role of the ASMA in vertical transport of pollutants during the monsoon season, including early autumn.
Source: *National Science Review*, DOI: 10.1093/nsr/nwz109

- **Rapid transport of East Asian pollution to the deep tropics:**
Demonstrates how cold surges during winter transport pollutants to the tropics and are further lifted by convection.
Source: *Atmospheric Chemistry and Physics*, DOI: 10.5194/acp-15-3565-2015

- **Wintertime Transport of Reactive Trace Gases From East Asia Into the Deep Tropics:**
Examines the role of cold surges and tropical convection in transporting East Asian pollution during winter months.
Source: *Journal of Geophysical Research: Atmospheres*, DOI: 10.1002/2017JD028231

We have included above references in the revised description.

- **Comment (Figure 9):** Remove this figure and its discussion as Figures 10 and 11 suffice.
    - **Response:** Thank you for your suggestion. However, we believe that Figure 9 provides critical context for the analyses presented in Figures 10 and 11. Specifically, Figure 9 illustrates the locations of elevated CO where the back trajectory calculations were initiated (represented by cross signs) and the clustering of these back trajectories. These clusters are essential in understanding the spatial distribution and source regions of elevated CO events. Furthermore, the trajectory clusters shown in Figure 9 serve as the basis for the gridded distribution of trajectory densities (frequencies) analyzed in Figures 10 and 11. By visualizing the initial locations and cluster patterns, Figure 9 offers valuable insights that help interpret the subsequent density distributions and their spatial relationships. We respectfully request to retain Figure 9 as it provides foundational information that enhances the interpretation and understanding of the results presented in Figures 10 and 11.

- **Comment (Section 3.5):** I am not sure this section is needed, but if it is, it seems like it belongs closer to Figure 5.
    - **Response:** Thank you for your comment. Section 3.5 presents a detailed analysis of back trajectory modeling for both elevated and low CO concentrations, derived from CO profiles measured in the atmospheric boundary layer over Taipei. These measurements, obtained from the dense flight operations illustrated in Figure 2, provide a comprehensive dataset that has not been previously reported. The analysis presented in Section 3.5 is essential as it leverages the extensive measurement profiles to minimize the influence of local pollution sources and instead focus on the transport pathways and origins of air masses with elevated and low CO concentrations. By examining measurements above ground level, we can better assess the sources and transport history of air masses enriched in CO and those with relatively low CO levels traveling to the industrial areas near Taipei City. Given the distinct focus and analytical depth of this section, we believe it is appropriately positioned within the manuscript. However, we are open to further discussion on repositioning it if deemed necessary for better coherence with Figure 5.
- **Comment (Line 384):** There is no plot in this manuscript that shows a clear and careful trend analysis.

  Response: Thank you for your comment. As noted in our previous response, a detailed trend analysis using NOAA flask measurements has been conducted in a separate manuscript (https://egusphere.copernicus.org/preprints/2024/egusphere-2024-2414/). To further enhance the current manuscript, we have revised Figure 7 to provide a statistical comparison of CO measurements from the NASA GTE experiments and IAGOS data. The revised figure includes box plots that present the mean, maximum, minimum, and the 25th, 50th (median), and 75th percentiles of CO concentrations for corresponding seasons (months).The comparison reveals the following insights. **PEM-West A (1991):** The mean CO concentration is approximately 100 ppbv, with occasional elevated values exceeding 200 ppbv at altitudes below 4 km. **IAGOS Measurements (2012):** The mean CO concentration exceeds 100 ppbv, with elevated values frequently surpassing 200 ppbv throughout the troposphere. These findings provide valuable context for understanding temporal changes in CO concentrations over the past two decades and highlight the differences between the historical NASA GTE experiments and the more recent IAGOS observations. By incorporating these statistical

comparisons, the revised manuscript offers a clearer representation of CO trends in the troposphere.

- **Comment (Line 389):** Do you have a citation for that statement? Can you compare the magnitude of both trends?

  Response: Thank you for your comment. We have revised the manuscript to include relevant references supporting the statement. Specifically, for comparisons of CO profiles downstream of Asian industrial areas, Figure 7 now provides a statistical comparison of in-situ CO measurements collected between 1991 and 2012 from the NASA GTE experiments and IAGOS data. These comparisons reveal an increase in CO concentrations in the upper troposphere over the North Pacific, with mean concentrations rising from approximately 100 ppbv in 1991 to values frequently exceeding 100 ppbv in 2012, with occasional peaks above 200 ppbv.

  For further analysis of CO trends in the upper troposphere, we have cited our separate study published at https://doi.org/10.5194/egusphere-2024-2414, which provides a detailed investigation into long-term trends upstream of Asian industrial areas, emphasizing the influence of anthropogenic emissions and atmospheric transport mechanisms.

  We believe these additions provide a more comprehensive context for the trends and offer a clearer comparison of their magnitudes across different time periods and regions.

- **Comment:** There is no data availability statement.
  - **Response:** A data availability statement has been added to the manuscript.

Editorial Requirements:

1. **LaTeX Template:** We have reformatted the manuscript using the PLOS LaTeX template.
2. **Language Editing:** The manuscript has been thoroughly edited for language and grammar using ChatGPT-4, a paid AI-based language model developed by OpenAI. I have invested in this professional tool to ensure the revised manuscript meets the highest standards of clarity, grammar, and precision. The tool was specifically utilized for language refinement and improving the readability of the

text, while all scientific content, interpretations, and conclusions remain solely the responsibility of the authors. The final version of the manuscript has been thoroughly reviewed to ensure its accuracy and appropriateness for submission.

3. **Financial Disclosure:** The "Role of Funder" statement has been updated to clarify that funders had no role in the study design, data collection, analysis, or decision to publish.
4. **Data Sharing Plan:** The data availability statement has been updated to indicate readiness for sharing upon acceptance.

We hope the revisions meet the expectations of the reviewers and the editorial team. Please let us know if further adjustments are required.

Sincerely, Kuo-Ying Wang
