# Supplementary material for: Elevated carbon monoxide observations over the North Pacific upper troposphere (July 2012–February 2013)
Source: PLoS One. 2025 May 21;20(5):e0323460. doi: 10.1371/journal.pone.0323460 (PMC12094778; doi:10.1371/journal.pone.0323460)
Supplement: Attachment — Submitted filename: replies_ref2_combined.pdf [file pone.0323460.s002.pdf]

Point-by-Point Replies to Reviewer 2

We sincerely thank the reviewer for the very insightful comments, which have significantly increased the clarity and overall quality of our revised manuscript.

We have read reviewer’ s comments carefully and revise our manuscript accordingly. We have paid specific attention to the request to highlight the differences in our study vs other studies in the past. We have put our work into the context of previous scientific perspective. We have explained how our work differs/confirms the previous studies cited by the reviewer.

Below, please find our detailed, point-by-point responses.

General Comments

Reviewer Comment:

Overall, the manuscript still currently does not describe how their analysis is novel and different from previous work.

Revised Reply:

The detailed analysis of IAGOS Pacific flights and the spatial distribution of CO measurements over the open ocean of the North Pacific upper troposphere, between 120°E (eastern Eurasia continent and the beginning of the North Pacific atmosphere) and 120°W (western edge of North America, end of the North Pacific atmosphere), during an 8-month period (July 2012 to February 2013) has not been previously reported. These measurements taken during this 8-month period over such a wide-spread of the North Pacific upper troposphere can only be made possible by the long-hull flights using A340-300 airplane to across North Pacific. Hence, these in-situ flight measurements are rare to characterize long-range transport of CO from Asia

to the downwind of the North Pacific upper troposphere.

Barret et al. (2025) used IAGOS data to validate satellite (IASI-A, IASI-B, and IASI-C; Infrared Atmospheric Sounding Interferometer) CO measurements, covering the period 2008 to 2019. They presented results from Taipei (25.09° N, 121.24° E) and Nagoya (34.85° N,136.81° E), located over the Northwestern Pacific, to verify the IAGOS measurements. Barret et al. (2025) showed time-series results of CO in the troposphere between the surface – 600 hPa layer and the 600 hPa – 200 hPa layer over Taipei City for

two periods: July 2012 – February 2013 and 2015 – mid 2018. Additional data were available from container measurements made by CARIBIC.

Lebourgeois et al. (2024) used IAGOS measurements from 2002 to 2019, the FLEXPART trajectory model, and surface CO emissions inventories to attribute the ground-level sources of elevated CO measured by IAGOS on a global scale. Elevated CO was linked to surface emissions (anthropogenic, biomass burning, or a combination of both) by analyzing the 20-day backtrajectory from each elevated CO measurement. In the East Asia upper troposphere—defined as 90° E to 150° E and 15° N to 45° N—elevated CO levels (175 ppb) occurred during the summer months, compared to 130 ppb in winter. During summer (June, July, August), about 95% of elevated CO originated from anthropogenic emissions, with the remainder from biomass burning and mixed sources. In winter (December, January, February), approximately 90% of elevated CO was attributed to anthropogenic sources, with the remainder from biomass burning and mixed emissions.

Cohen et al. (2018) discussed the climatology and long-term evolution of ozone and carbon monoxide in the upper troposphere – lower stratosphere (UTLS) at northern midlatitudes, as observed by IAGOS from 1995 to 2013 on a global scale. In the East Asia region, defined as 105° E – 145° E and 30° N – 50° N, elevated CO levels were found in the upper troposphere during late spring (May) and summer (June and July), while the lower stratosphere exhibited elevated CO levels during the summer (June, July, August). Cohen et al. (2018) also reported negative CO trends (5th percentile, mean value, and 95th percentile) over the 105° E – 145° E and 30° N – N50° N area of the East Asia upper troposphere. It is noteworthy that IAGOS Pacific measurements commenced in July 2012 using China Airlines' in-service A340-300 aircraft.

Tsivlidou et al. (2023) employed FLEXPART 20-day backtrajectories based on elevated CO measured by IAGOS over China and the tropical West Pacific (25° N – 25° S) during 2002 – 2020 to attribute the sources of elevated CO. The methodology used was similar to that of Lebourgeois et al. (2024). Again, IAGOS Pacific measurements were initiated in July 2012.

The aforementioned studies utilized extensive data periods (2008 – 2019, 2002 – 2019, 1995 – 2013, 2002 – 2020) from IAGOS measurements, covering areas primarily over the Atlantic, Western Europe, North America, South America, and Africa, as well as some flights from Europe to Japan, China, and Korea. In contrast, the IAGOS Pacific measurements, started in July 2012, were conducted over the open ocean of the North Pacific (120° E – 120° W) for flights from East Asia to North America.

Previous studies using IAGOS data have provided a broad, seasonal (summer, winter) view of CO characteristics over the eastern edge of the Eurasian continent and at the intersection of Eurasia and the northwestern Pacific. In this work, we complement these long-term and seasonal (summer, winter) studies by providing a detailed, zoomed-in analysis on a day-to-day and month-to-month basis for the first 8 months of IAGOS Pacific flights across the open ocean of the North Pacific upper troposphere. This detailed analysis captures episodic events that are missed in seasonal data and reveals transient CO peaks critical for understanding episodic pollution transport. We focus on July 2012 – February 2013, marking the initiation of IAGOS Pacific flights, which provides a baseline for subsequent trends, supported by a sufficient data density for detailed analysis. Future work may explore later years to assess temporal evolution.

We present the distribution of elevated CO over the North Pacific open ocean upper troposphere and conduct backtrajectory calculations to identify the sources of both elevated and low CO. For the lower troposphere, we analyze a total of 520 CO profiles over Taipei (at a vertical resolution of 50 m), performing profile-by-profile backtrajectory analysis for both elevated and low CO measurements. The comparisons reveal distinct sources of air masses arriving in Taipei, complementing the upper tropospheric focus on sources of elevated versus low CO.

Reviewer Comment:

There are many papers on deep convection transporting pollution to the upper troposphere over Asia

Revised Reply:

We have included the following description in the introduction:

Asian anthropogenic emissions and boreal biomass burning dominate CO in the North Pacific upper troposphere (Jaffe et al., 1999). Fast uplift via convection and mid-latitude cyclones facilitates CO injection into the upper troposphere (Jiang et al., 2007; Randel et al., 2010). Spring and early summer show heightened CO due to efficient vertical transport and favorable wind patterns (e.g., the Westerlies). Satellite observations (Jiang et al., 2007), in situ aircraft observations (Cohen et al., 2018; Lebourgeois et al., 2024; Barret et al., 2025), and models such as HYSPLIT (Chen et al., 2021), FLEXPART (Tsivlidou et al., 2023), and GEOS-Chem (Liang et al., 2004; Miyazaki et al., 2020) are frequently employed to study the long-range transport of air pollutants from Asian anthropogenic and

biomass burning emissions. While Jiang et al. (2007) used satellite data to demonstrate seasonal CO transport, our IAGOS profiles provide in situ validation with finer temporal detail over the North Pacific and allow refined source attribution via backtrajectory analysis.

Reviewer Comment (related to citation examples):

e.g., \url{https://www.science.org/doi/full/10.1126/science.1182274}

Revised Reply:

Randel et al. (2010) used the Atmospheric Chemistry Experiment Fourier Transform Spectrometer (ACE-FTS) and MLS satellite measurements of atmospheric hydrogen cyanide (HCN) along with the three-dimensional climate model WACCM3 to study elevated HCN in the lower stratosphere during 2004 – 2009 over regions impacted by the Asian summer monsoon. Their results demonstrate that surface air enriched in HCN is rapidly transported into the stratosphere by deep convection. They also noted that, in addition to the maximum vertical transport during boreal summer (June – August), seasonally varying HCN sources include biomass burning over Indonesia and Africa during boreal spring (March – May) and over Africa and South America during austral spring (September – November), with deep convection facilitating the transport of these emissions into the upper troposphere.

Reviewer Comment:

The paper currently reads that the presented analysis is the 'first' to show this, which is incorrect. Much more discussion of previous studies and how this work is novel is needed.

Revised Reply:

We have removed all descriptive words such as "first" from the revised manuscript. Our work is positioned as an extension of previous missions and studies. We have incorporated the references provided by the reviewer and clarified the novelty of our approach by emphasizing the high temporal resolution (day-to-day and month-to-month) analysis of IAGOS Pacific flights during the initial 8-month period, which captures transient CO peaks that longer-term seasonal studies may overlook.

Reviewer Comment:

The authors must better describe why they only look at data from 2012-2013. There is much more data than this available from IAGOS.

Revised Reply:

Barret et al. (2025) used IAGOS data to validate satellite (IASI-A, IASI-B, and IASI-C; Infrared Atmospheric Sounding Interferometer) CO data, covering 2008 – 2019. They presented time-series results of CO in the troposphere for two periods: July 2012 – February 2013 over Taipei and during 2015 – mid 2018. Although longer datasets exist, we focused on July 2012 – February 2013 because it marks the initiation of IAGOS Pacific flights using a China Airlines Airbus A340-300. This period offers sufficient data density for a detailed, high-temporal resolution analysis and serves as an important baseline for subsequent trend analysis. Future work could extend this approach to additional years to assess temporal evolution.

Reviewer Comment:

Why compare their data to previous NASA aircraft missions, but not other years of IAGOS data? The authors also need to better describe how their work is different from other papers on IAGOS CO trends that cover longer time periods.

Revised Reply:

1. **Comparison with Previous Studies:**
   Lebourgeois et al. (2024) used IAGOS 2002 – 2019 measurements, the FLEXPART trajectory model, and surface CO emissions inventories to attribute sources of elevated CO on a global scale. They found that in the East Asia upper troposphere (defined between 90°E and 150°E and 15°N – 45°N), elevated CO concentrations (175 ppb) occur in the summer months compared to 130 ppb in winter, with anthropogenic emissions dominating during summer and biomass burning contributing more during winter.
   Cohen et al. (2018) analyzed the long-term evolution of ozone and carbon monoxide in the upper troposphere – lower stratosphere from 1995 to 2013 and identified seasonal trends in the East Asia region (105°E – 145°E

and 30°N – 50°N). They reported negative trends in CO. Tsivlidou et al. (2023) applied FLEXPART backtrajectory analysis to elevated CO measured by IAGOS over China and the tropical western Pacific during 2002 – 2020. Their method is similar to that used by Lebourgeois et al. (2024).

2. **Our Work's Novelty:**
   In contrast to these studies that provide a zoomed-out view on seasonal or decadal trends, our work provides a detailed, high-temporal resolution (day-to-day and month-to-month) analysis of the first 8 months (July 2012 – February 2013) of IAGOS Pacific flights. This focused period allows us to capture episodic events and transient CO peaks that are often averaged out in longer-term studies. By doing so, we can better understand the mechanisms of episodic pollution transport and provide a baseline for future trend analyses.

Reviewer Comment:

Also, there was no marked-up manuscript submitted as part of this revision, which made the changes difficult to assess.

Revised Reply:

We have provided a marked-up version of the revised manuscript for your convenience.

Specific Comments

Reviewer Comment (Line 45):

I do not know what the authors mean by "the first time". What about the MOZAIC flights, for example, mentioned in Section 2.2? Did none of the MOZAIC flights go over the Pacific? Is this really necessary to state at all? You even say this on line 82 "Two decades of MOZAIC measurements have affirmed the reliability of this instrument for continuous operation over 6-12 months aboard commercial passenger aircraft."

Revised Reply:

We have revised the wording and removed the term "first" from the revised manuscript to avoid any ambiguity. Our focus is on the specific characteristics of the IAGOS Pacific flights, not on asserting uniqueness relative to MOZAIC flights.

Reviewer Comment (Line 172):

Again, are these really the first?

Revised Reply:

We have removed the word "first" from the revised manuscript to accurately represent our findings without implying novelty that is not supported by the literature.

Reviewer Comment (Figure 5f):

Why are there blue box and whiskers?

Revised Reply:

We have revised the color scheme for Figure 5f to ensure consistency with the other plots. The updated figure now uses a uniform color palette that improves clarity and comparability across figures.

Reviewer Comment (Figure 6):

Why are these regions not identical to the regions in Figure 5? For example, Fig. 5f is 160 – 110°W, 75 – 25°S but Fig. 6e is 160 – 110°W, 70 – 20°N. I don't think the altitude trace is needed, just say the measurements are between 9 – 13.

Revised Reply:

Figure 5 displays measurements from all flights made by the IAGOS Package 1 on the CAL A340-300 B18316, whereas Figure 6 focuses on measurements over the Northern Hemisphere midlatitudes, particularly those from flights across the North Pacific. The altitude traces in Figure 6 indicate the data density per flight. We have now clarified this point in the revised manuscript by stating, "Figure 6 isolates Northern Hemisphere midlatitude data (e.g., 20°N – 70°N) to highlight North Pacific trends, with altitude traces retained to reflect data density per flight," and we have removed unnecessary altitude markers.

Reviewer Comment (Line 241):

I am again not convinced "first time" is really appropriate here.

Revised Reply:

We have removed the word "first" from the revised manuscript to avoid any potential misinterpretation regarding the novelty of our observations.

Reviewer Comment (Section 3.2.4):

This should read 'Comparisons With'

Revised Reply:

We have corrected the heading in Section 3.2.4 to read "Comparisons With" as suggested.

Reviewer Comment (Figure 7):

I would strongly suggest plotting similar time periods and locations on top of each other. You could plot, for example, the box-whisker from Fig. 7l and 7d on the same plot to better visualize differences.

Revised Reply:

We have rearranged the plots so that those representing similar time periods (by month) are placed adjacent to each other. For example, Figure 7d now shows measurements during September – October 2012 while Figure 7l shows measurements during February – April 2013. We have used identical x-axis ranges and added a highlighted dashed vertical line to facilitate direct comparisons between the figures.

Reviewer Comment (Line 283):

The suggestion above for Fig. 7 would make this point stronger.

Revised Reply:

We have added a vertical black dashed line at 200 ppbv in Figure 7 to underscore the shift in CO concentrations: in 1991, most values ranged from 50 to 150 ppbv (Figure 7b), whereas in 2012, values ranged from 50 to 200+ ppbv (Figure 7d). We have included this explanation in the caption of Figure 7.

Reviewer Comment (Figure 13):

What further insight does this provide that hasn't already been shown in Figs. 5 – 7?

Revised Reply:

Figure 13 presents high-time-resolution lower tropospheric profiles over Taipei, while Figures 5 – 7 focus on the upper tropospheric characteristics across the North Pacific. In addition, Figure 13 provides a profile-by-profile backtrajectory analysis of both elevated and low CO measurements over Taipei, demonstrating distinctive source regions of air masses. This detailed analysis complements the upper tropospheric focus of Figures 5 – 7 and highlights differences in source attribution between the upper and lower troposphere.

Extended Point-by-Point Replies to Reviewer 2

1 General Comments

Overall, the manuscript still currently does not describe how their analysis is novel and different from previous work.

Reply.

We have reviewed references kindly provided by the reviewer.

The detailed analysis of IAGOS Pacific flights and spatial distribution of CO measurements during the period of 8 months, from July 2012 to February 2013 over the North Pacific upper troposphere (between $120°E$ and $120°W$) were not reported in the references (see below for details).

Barret et al. (2025) used IAGOS data to validates satellite (IASI-A, IASI-B and IASI-C; Infrared Atmospheric Sounding Interferometer) CO data, covering 2008 to 2019. Results from Taipei ($25.09°N, 121.24°E$) and Nagoya ($34.85°N, 136.81°E$), located over Northwester Pacific, were presented to verified with the IAGOS measurements. Barret et al. (2025) showed time-series results of CO in the troposphere between surface-600 hPa and 600 hPa - 200 hPa over Taipei City during July 2012 - February 203; and during 2015 - mid 2018. More data were available from container measurements made by CARIBIC.

Lebourgeois et al. (2024) used IAGOS 2002-2019 measurements, the FLEXPART trajectory model, and surface CO emissions inventories to attribute ground-level source of elevated CO measured by the IAGOS on a global domain. The elevated CO measured was linked to surface emissions (anthropogenic, biomass burning, or both) by the 20-day backtrajectory from each elevated CO measurement. For the East Asia upper troposphere region, which is defined between $90°E$ and $150°E$ and between $15°N$ and $45°N$, elevated CO (175 ppb) occurred during the summer months than during the winter months (130 ppb). During the summer months (June, July, August), about 95% of elevated CO are from anthropogenic emission, and the rest are from biomass burning and mixed sources. During the winter months (December, January, February), about 90form anthropogenic sources, and the rest are from biomass and mixed emissions.

Cohen et al. (2018) discussed climatology and long-term evolution of ozone and carbon monoxide in the upper troposphere–lower stratosphere (UTLS) at northern midlatitudes, as seen by IAGOS from 1995 to 2013) on a global domain. The East Asia region, defined as $105°E - 145°E$ and $30°N - 50°N$, shows elevated CO over the East Asia upper troposphere during the late spring month of May and summer months of June and July. For the lower stratosphere, elevated CO levels are in the summer months (June, July, August). We note

that Cohen et al. (2018) calculated negative CO trends (5th percentile, mean value, and 95th percentile) over the $105°E - 145°E$ and $30°N - 50°N$ area of East Asia upper troposphere. We note that the IAGOS Pacific measurements were started in July 2012 with China Airlines commercial in-service A340-300 Airbus airplane.

Tsivlidou et al. (2023) used FLEXPART 20-day backtrajectories based on elevated CO measured by IAGOS over China and tropical west Pacific ($25°N$-$25°S$) during the period 2002-2020 to attribute sources of elevated CO. The method used in this work is the same as in Lebourgeois et al. (2024). Again, the IAGOS Pacific measurements were started in July 2012.

Extensive data periods (2008-2019, 2002-2019, 1995-2013, 2002-2020) from IAGOS measurements were used in above studies on areas mostly over the Atlantic, Western Europe, North America, South America, and Africa, and some flights from Europe to Japan, China, and Korea. The IAGOS Pacific measurements across the Pacific upper atmosphere were started in July 2012, over the open oceanic atmosphere of the North Pacific ($120°E - 120°W$) for flights from East Asia to North America.

Previous studies with IAGOS data provide a zoom-out views of the CO characteristic in the seasonal time scale (winter versus summer months) over the eastern edge of the Euro-Asia continent and between the intersection of the Euro-Asia continent and the northwestern Pacific.

In this work, we compliment above long-term and seasonal studies with zoom-in details of day-to-day and month-to-month of the first 8 months of the detailed IAGOS flights across open ocean of the North Pacific upper troposphere. The detailed zoom-in analysis capturing episodic events missed in seasonal data. Our daily/monthly resolution reveals transient CO peaks missed in seasonal analyses, critical for understanding episodic pollution transport. We focused on July 2012–February 2013 as it marks the initiation of IAGOS Pacific flights, offering a baseline for subsequent trends, with sufficient data density for detailed analysis. Later years could be explored in future work to assess temporal evolution.

We show the distribution of elevated CO over the North Pacific open ocean upper troposphere, conduct backtrajectory calculations, identify sources of the elevated CO over the open ocean of the North Pacific upper troposphere. In comparison, We have also conduct backtrajectory calculations of low CO over the open ocean of the North Pacific upper troposphere. While both elevated and low CO were measured over the open ocean of the North Pacific upper troposphere, backtrajectory calculations reveal distinctive source of airs when compare elevated CO with the low CO measurements. For the lower troposphere, we show a total of 520 profiles of CO measurements over Taipei (at a vertical resolution of 50m), profile by profile, and conduct backtrajectory analysis of elevated and low CO measurements. The

comparisons show distinctive source of airs traveling to Taipei when compare elevated CO with the low CO measurements, complementing the upper tropospheric focus of sources of elevated versus low CO.

There are many papers on deep convection transporting pollution to the upper troposphere over Asia

Reply. We have included following description in the introduction:

Asian anthropogenic emissions and boreal biomass burning dominate CO in the North Pacific upper troposphere (Jaffe et al., 1999). Fast uplift via convection and mid-latitude cyclones facilitates CO injection into the upper troposphere, enabling long-range transport (Jiang et al., 2007; Randel et al., 2010). Spring and early summer show heightened CO due to efficient vertical transport and favorable wind patterns (e.g., Westerlies). Satellite observations (Jiang et al., 2007), in-situ airplane observations (Cohen et al. (2018); Lebourgeois et al. (2024); Barret et al., 2025), and models such as HYSPLIT (Chen et al.,2021), FLEXPART (Tsivlidou et al., 2023), and GEOS-Chem (Liang et al., 2004; Miyazaki et al., 2020) are frequently employed to study long-range transport of air pollutants from Asian anthropogenic and biomass burning emissions. While Jiang et al. (2007) used satellite data to show seasonal CO transport, our IAGOS profiles provide in-situ validation and finer temporal detail over the North Pacific, refining source attribution via backtrajectories.

(eg., `https://www.science.org/doi/full/10.1126/science.1182274`,

Reply. Randel, W.J., Park, M., Emmons, L., Kinnison, D., Bernath, P., Walker, K.A., Boone, C. and Pumphrey, H., 2010. Asian monsoon transport of pollution to the stratosphere. Science, 328(5978), pp.611-613. Reply. Randel et al. (2010) used and the Atmospheric Chemistry Experiment Fourier Transform Spectrometer (ACE-FTS) satellite instrument, and the MLS satellite measurements of atmospheric hydrogen cyanide (HCN) and a three-dimensional climate model WACCM3 to study elevated HCN observed in the lower stratosphere during 2004-2009 over the region where the Asian summer monsoon were active.

Randel et al. (2010) found that surface air, riched in HCN, is transported from the surface to deep within the stratosphere by deep convection processes during the Asian monsoon months. In addition to the maximum vertical transport associated with the Asian monsoon during boreal summer (June, July, August), seasonally varying sources of HCN include burning over Indonesia and Africa during boreal spring (March to May), and burning over Africa and South America during austral spring (September to November), with these emissions transported to the upper troposphere by deep convection.

`https://agupubs.onlinelibrary.wiley.com/doi/full/10.1029/2007GL030638` and many others).

Reply. Jonathan H. Jiang, Nathaniel J. Livesey, Hui Su, Lori Neary, John C. McConnell,

Nigel A. D. Richards First published: 27 September 2007 https://doi.org/10.1029/2007GL030638

Jiang, J.H., Livesey, N.J., Su, H., Neary, L., McConnell, J.C. and Richards, N.A., 2007. Connecting surface emissions, convective uplifting, and long-range transport of carbon monoxide in the upper troposphere: New observations from the Aura Microwave Limb Sounder. Geophysical Research Letters, 34(18).

Jiang et al. (2007) showed two years of upper-tropospheric (UT) carbon monoxide (CO) measurements from the Aura Microwave Limb Sounder, analyzed alongside surface CO emission climatology and NCEP analyses (winds, outgoing longwave radiation, and vertically integrated water content). Jian et al. (2007) showed distinct spatial patterns, temporal variations, and long-range transport associated with the elevated CO over the North Pacific upper troposphere. The results indicate that UT CO levels peak over Southeast Asia during boreal summer, primarily due to anthropogenic emissions lifted by convection. Long-range transport carries Asian anthropogenic CO emissions across the Pacific Ocean to North America, significantly impacting global air quality, especially during summer months.

Jiang et al. (2007) found elevated CO over the Northwest $(20°N - 45°N, 140°E - 170°E)$ and Northeast $(20°N - 45°N, 170°W - 140°W)$ Pacific upper troposphere during the summer months (June, July, August).

The paper currently reads that the presented analysis is the 'first' to show this, which is incorrect. Much more discussion of previous studies and how this work is novel is needed.

Reply. We have removed all the descriptive word "first" in the revised manuscript. Our work is an extension of previous previous missions and studies. We have included references provided by the reviewer in the revised manuscript.

The authors must better describe why they only look at data from 2012-2013. There is much more data than this available from IAGOS `https://amt.copernicus.org/articles/18/129/2025/`.

Reply. Barret, B., Loicq, P., Le Flochmoën, E., Bennouna, Y., Hadji-Lazaro, J., Hurtmans, D., and Sauvage, B.: Validation of 12 years (2008–2019) of IASI-A CO with IAGOS aircraft observations, Atmos. Meas. Tech., 18, 129–149, `https://doi.org/10.5194/amt-18-129-2025`, 2025.

Barret et al. (2025) used IAGOS data to validates satellite (IASI-A, IASI-B and IASI-C; Infrared Atmospheric Sounding Interferometer) CO data, covering 2008 to 2019. Results from Taipei $(25.09°N, 121.24°E)$ and Nagoya $(34.85°N, 136.81°E)$, located over Northwester Pacific, were presented to verified with the IAGOS measurements. Barret et al. (2025) showed time-series results of CO in the troposphere between surface-600 hPa and 600 hPa - 200 hPa over Taipei City during July 2012 - February 203; and during 2015 - mid 2018. More data were available from container measurements made by CARIBIC.

Why compare their data to previous NASA aircraft missions, but not other years of IAGOS data? The authors also need to better describe how their work is different from other papers on IAGOS CO trends that cover longer time periods. See the following (non-comprehensive) list of papers:

1. https://acp.copernicus.org/articles/24/13975/2024/acp-24-13975-2024.html Also discusses elevated CO over East Asia.

Reply. Lebourgeois, T., Sauvage, B., Wolff, P., Josse, B., Marécal, V., Bennouna, Y., Blot, R., Boulanger, D., Clark, H., Cousin, J.-M., Nedelec, P., and Thouret, V.: Seasonal, regional, and vertical characteristics of high-carbon-monoxide plumes along with their associated ozone anomalies, as seen by IAGOS between 2002 and 2019, Atmos. Chem. Phys., 24, 13975–14004, https://doi.org/10.5194/acp-24-13975-2024, 2024.

Lebourgeois et al. (2024) used IAGOS 2002-2019 measruemnts, the FLEXPART trajectory model, and surface CO emissions inventories to attribute ground-level source of elevated CO measured by the IAGOS on a global domain. The elevated CO measured was linked to surface emissions (anthropogenic, biomass burning, or both) by the 20-day backtrajectory from each elevated CO measurement. For the East Asia upper troposphere region, which is defined between $90°E$ and $150°E$ and between $15°N$ and $45°N$, elevated CO (175 ppb) occurred during the summer months than during the winter months (130 ppb). During the summer months (June, July, August), about 95% of elevated CO are from anthropogenic emission, and the rest are from biomass burning and mixed sources. During the winter months (December, January, February), about 90form anthropogenic sources, and the rest are from biomass and mixed emissions.

2. https://acp.copernicus.org/articles/18/5415/2018/ Also discusses CO seasonality and elevated CO over East Asia.

Reply. Cohen, Y., Petetin, H., Thouret, V., Marécal, V., Josse, B., Clark, H., Sauvage, B., Fontaine, A., Athier, G., Blot, R., Boulanger, D., Cousin, J.-M., and Nédélec, P.: Climatology and long-term evolution of ozone and carbon monoxide in the upper troposphere–lower stratosphere (UTLS) at northern midlatitudes, as seen by IAGOS from 1995 to 2013, Atmos. Chem. Phys., 18, 5415–5453, https://doi.org/10.5194/acp-18-5415-2018, 2018.

Cohen et al. (2018) discussed climatology and long-term evolution of ozone and carbon monoxide in the upper troposphere–lower stratosphere (UTLS) at northern midlatitudes, as seen by IAGOS from 1995 to 2013) on a global domain.

The East Asia region, defined as $105°E-145°E$ and $30°N-50°N$, shows elevated CO over the East Asia upper troposphere during the late spring month of May and summer months of June and July. For the lower stratosphere, elevated CO levels are in the summer months (June, July, August). We note that Cohen et al. (2018) calculated negative CO trends (5th

percentile, mean value, and 95th percentile) over the $105°E - 145°E$ and $30°N - 50°N$ area of East Asia upper troposphere.

3. https://acp.copernicus.org/articles/23/14039/2023/acp-23-14039-2023.pdf Also discusses CO seasonality and highlights the impact of anthropogenic emissions.

Reply.

Tsivlidou, M., Sauvage, B., Bennouna, Y., Blot, R., Boulanger, D., Clark, H., Le Flochmoën, E., Nédélec, P., Thouret, V., Wolff, P., and Barret, B.: Tropical tropospheric ozone and carbon monoxide distributions: characteristics, origins, and control factors, as seen by IAGOS and IASI, Atmos. Chem. Phys., 23, 14039–14063, https://doi.org/10.5194/acp-23-14039-2023, 2023.

Tsivlidou et al. (2023) used FLEXPART 20-day backtrajectories based on elevated CO measured by IAGOS over China and tropical west Pacific ($25°N$-$25°S$) during the period 2002-2020 to attribute sources of elevated CO. The method used in this work is the same as in Lebourgeois et al. (2024).

As shown in reply above. Extensive data periods (2008-2019, 2002-2019, 1995-2013, 2002-2020) from IAGOS measurements were used in above studies on areas mostly over the Atlantic, Western Europe, North America, South America, and Africa, and some flights from Europe to Japan, China, and Korea. The IAGOS Pacific measurements across the Pacific upper atmosphere were started in July 2012, over the open oceanic atmosphere of the North Pacific ($120°E - 120°W$) for flights from East Asia to North America.

Previous studies with IAGOS data provide a zoom-out views of the CO characteristic in the seasonal time scale (winter versus summer months) over the western edge of the Euro-Asia continent and between the intersection of the Euro-Asia Continent and the northwestern Pacific.

In this work, we compliment above long-term and seasonal studies with zoom-in details of day-to-day and month-to-month of the first 8 months of the detailed IAGOS flights across open ocean of the North Pacific upper troposphere. We show the distribution of elevated CO over the North Pacific open ocean upper troposphere, conduct backtrajectory calculations, identify sources of the elevated CO over the open ocean of the North Pacific upper troposphere. In comparison, We have also conduct backtrajectory calculations of low CO over the open ocean of the North Pacific upper troposphere. While both elevated and low CO were measured over the open ocean of the North Pacific upper troposphere, backtrajectory calculations reveal distinctive source of airs when compare elevated CO with the low CO measurements. For the lower troposphere, we show a total of 520 profiles of CO measurements over Taipei (at a vertical resolution of 50m), profile by profile, and conduct backtrajectory analysis of elevated and low CO measurements. The comparisons show distinctive source of

airs traveling to Taipei when compare elevated CO with the low CO measurements.

Our daily/monthly resolution reveals transient CO peaks missed in seasonal analyses, critical for understanding episodic pollution transport. We focused on July 2012–February 2013 as it marks the initiation of IAGOS Pacific flights, offering a baseline for subsequent trends, with sufficient data density for detailed analysis. Later years could be explored in future work to assess temporal evolution.

Also, there was no marked up manuscript submitted as part of this revision, which made the changes difficult to assess.

Reply. We have provided marked up version of the revised manuscript.

2 Specific Comments

Line 45 – I do not know what the authors mean by "the first time". What about the MOZAIC flights for example mentioned in Section 2.2? Did none of the MOZAIC flights go over the Pacific? Is this really necessary to state at all? You even say this on line 82 "Two decades of MOZAIC measurements have affirmed the reliability of this instrument for continuous operation over 6-12 months aboard commercial passenger aircraft."

Reply. We have revised the wording, and removed "first" from the revised manuscript.

Line 172 – Again are these really the first?

Reply. We have removed the word "first" from the revised manuscript.

Figure 5f – Why are there blue box and whiskers?

Reply. We have revised the color for Figure 5f to be consistent with other plots.

Figure 6 – Why are these regions not identical to the regions in Figure 5? Example, Fig. 5f is 160-110W, 75-25S but Fig. 6e is 160-110W, 70-20N. I don't think the altitude trace is needed, just say the measurements are between 9-13

Reply. Figure 5 shows measurements all measurements made by the IAGOS Package 1 onboard CAL airplane A340-300 B18316. Figure 6 focus on measurements over the Northern Hemisphere midlatitude, especially across the open oceanic atmosphere upper troposphere of the North Pacific, between East Asia and North America. The altitude data shows amounts of data from each flight used in the analysis. We have included following description in the revised manuscript "Figure 6 isolates Northern Hemisphere midlatitude data (e.g., $20°N\breve{~}70°N$) to highlight North Pacific trends, with altitude traces retained to reflect data density per flight."

Line 241 – I am again not convinced "first time" is really appropriate here.

Reply. We have removed the word "first" from the revised manuscript.

Section 3.2.4 – This should read 'Comparisons With'

Reply. We have corrected the words to "Comparisons With".

Figure 7 – I would strongly suggest plotting similar time periods and locations on top of each other. You could plot for example the box whisker from Fig. 7l and 7d on the same plot to better visualize differences.

Reply. We have arranged the plots to be close in time (month) for comparison. Figure 7d shows measurements during Sep-Oct (2012), while Figure 7l shows measurements during Feb-Apr (2001). Hence, We put Figure 7h, which shows measurements during Feb (2013), above Figure 7l for a comparison close in time (Feb versus Feb-Apr). We have used the same ranges in x-axis, with a highlighted x value in dashed line to enable the comparison between figures.

Line 283 – The suggestion above for Fig. 7 would make this point stronger.

Reply. We have used a vertical black dashed line highlights CO concentrations at 200 ppbv to reveal the changes in profiles of CO measurements. In 1991, most CO concentrations ranged from 50 ppbv to 150 ppbv (Fig. 7b), whereas in 2012, concentrations ranged from 50 ppbv to 200 ppbv (Fig. 7d). The upper troposphere in 2012 showed increased pollution, with CO concentrations exceeding 200 ppbv, compared to concentrations below 150 ppbv in 1991. In the lower troposphere (below 3 km), maximum CO concentrations increased from approximately 700 ppbv in 1991 to 780 ppbv in 2012. These trends highlight a marked increase in CO levels throughout the troposphere, especially in the upper troposphere. We have included following description in the caption of Figure 7: A dashed line at 200 ppbv underscores a shift from 50–150 ppbv in 1991 to 50–200+ ppbv in 2012, indicating enhanced upper tropospheric pollution.

Figure 13 – What further insight does this provide that hasn't already been shown in Fig. 5-7?

Reply. In this work, we compliment above long-term and seasonal studies with zoom-in details of day-to-day and month-to-month of the first 8 months of the detailed IAGOS flights across open ocean of the North Pacific upper troposphere. We show the distribution of elevated CO over the North Pacific open ocean upper troposphere, conduct backtrajectory calculations, identify sources of the elevated CO over the open ocean of the North Pacific upper troposphere. In comparison, We have also conduct backtrajectory calculations of low CO over the open ocean of the North Pacific upper troposphere. While both elevated and low CO were measured over the open ocean of the North Pacific upper troposphere, backtrajectory calculations reveal distinctive source of airs when compare elevated CO with the low CO measurements (shown in Figures 5-12). For the lower troposphere, we show a total of 520 profiles of CO measurements over Taipei (at a vertical resolution of 50m), profile by profile, and conduct backtrajectory analysis of elevated and low CO measurements. The

comparisons show distinctive source of airs traveling to Taipei when compare elevated CO with the low CO measurements (shown in Figures 13-14). As such, Figure 13 presents high-time resolution lower tropospheric profiles over Taipei, while Figure 14 reveals distinct source regions for elevated versus low CO, complementing the upper tropospheric focus of Figures 5–7.
